# Supplementary material for: MetaRibo-Seq measures translation in microbiomes
Source: Nat Commun. 2020 Jun 29;11:3268. doi: 10.1038/s41467-020-17081-z (PMC7324362; doi:10.1038/s41467-020-17081-z)
Supplement: Supplementary file 10 — Supplementary Data 7 [file 41467_2020_17081_MOESM10_ESM.zip › File2/Confidence_High_Taxonomy/425619_out.krona.html]

Javascript must be enabled to view this page.

members
magnitude
magnitudeUnassigned
count
unassigned
taxon
rank

425619\_out

114

114
superkingdom
2

phylum
976
114

114
class
200643

171549
order
114

1
2005525
family

genus
375288
1

823

SRS077849\_contig\_number\_4691
species
1

113
815
family

genus

SRS053398\_contig\_number\_873SRS063518\_contig\_number\_22119
2
816
113

51
2292944

SRS011302\_contig\_number\_contig-100\_233.139374SRS012969\_contig\_number\_702SRS013382\_contig\_number\_547SRS014459\_contig\_number\_7415SRS015065\_contig\_number\_34153SRS015369\_contig\_number\_1413SRS016056\_contig\_number\_583SRS016629\_contig\_number\_401SRS016954\_contig\_number\_4358SRS017433\_contig\_number\_168SRS018427\_contig\_number\_25711SRS018836\_contig\_number\_13876SRS019445\_contig\_number\_18846SRS020328\_contig\_number\_18726SRS023526\_contig\_number\_10274SRS024625\_contig\_number\_7923SRS042966\_contig\_number\_8175SRS043768\_contig\_number\_1027SRS045195\_contig\_number\_2941SRS047433\_contig\_number\_15881SRS050026\_contig\_number\_732SRS050801\_contig\_number\_2006SRS050941\_contig\_number\_6359SRS058770\_contig\_number\_9785SRS062701\_contig\_number\_5809SRS063370\_contig\_number\_4700SRS064557\_contig\_number\_21766SRS064645\_contig\_number\_209SRS076976\_contig\_number\_7141SRS077294\_contig\_number\_15821SRS077552\_contig\_number\_17697SRS078242\_contig\_number\_19710SRS078665\_contig\_number\_958SRS100021\_contig\_number\_9762SRS1041037\_contig\_number\_576SRS1041139\_contig\_number\_596SRS1041142\_contig\_number\_1440SRS1041157\_contig\_number\_contig-100\_1004.52326SRS1055038\_contig\_number\_5416SRS1055056\_contig\_number\_4403SRS142980\_contig\_number\_6562SRS143895\_contig\_number\_2880SRS143991\_contig\_number\_16353SRS148196\_contig\_number\_43243SRS148874\_contig\_number\_9844SRS150029\_contig\_number\_13797SRS892882\_contig\_number\_10046SRS893252\_contig\_number\_18905SRS893341\_contig\_number\_3232SRS893373\_contig\_number\_18571SRS971275\_contig\_number\_46062
species

1
47678
species

SRS044535\_contig\_number\_8479

species

SRS054352\_contig\_number\_15961SRS103987\_contig\_number\_14180SRS1041164\_contig\_number\_1307
821
3

1
species

SRS104975\_contig\_number\_14159
2292939


SRS019161\_contig\_number\_1205SRS052697\_contig\_number\_3683SRS893327\_contig\_number\_7981
species
1262754
3

species

SRS013521\_contig\_number\_10275SRS013800\_contig\_number\_3833SRS014235\_contig\_number\_5256SRS015217\_contig\_number\_7676SRS015579\_contig\_number\_560SRS016989\_contig\_number\_971SRS017375\_contig\_number\_823SRS017821\_contig\_number\_12107SRS019685\_contig\_number\_12410SRS019910\_contig\_number\_23329SRS020233\_contig\_number\_15996SRS020394\_contig\_number\_4563SRS021948\_contig\_number\_14104SRS024663\_contig\_number\_13833SRS045004\_contig\_number\_19825SRS045645\_contig\_number\_5575SRS047014\_contig\_number\_729SRS049995\_contig\_number\_7610SRS051031\_contig\_number\_33254SRS051882\_contig\_number\_1317SRS052027\_contig\_number\_16708SRS053087\_contig\_number\_2151SRS076756\_contig\_number\_2489SRS078176\_contig\_number\_444SRS101376\_contig\_number\_6979SRS1041091\_contig\_number\_20245SRS1041133\_contig\_number\_27012SRS1055043\_contig\_number\_16184SRS1055067\_contig\_number\_16561SRS1055083\_contig\_number\_270SRS1055095\_contig\_number\_2222SRS142890\_contig\_number\_contig-100\_844.214902SRS146764\_contig\_number\_2857SRS148319\_contig\_number\_436SRS893172\_contig\_number\_10910SRS893187\_contig\_number\_10627SRS893369\_contig\_number\_8832SRS893383\_contig\_number\_contig-100\_1324.180843
204516
38

2292941

SRS011134\_contig\_number\_37148SRS013638\_contig\_number\_11377SRS014683\_contig\_number\_16695SRS019030\_contig\_number\_3819SRS022609\_contig\_number\_9960SRS050974\_contig\_number\_1371SRS1041036\_contig\_number\_1223SRS1041095\_contig\_number\_1402SRS1041144\_contig\_number\_8275SRS1041147\_contig\_number\_2183SRS1055099\_contig\_number\_15021SRS144603\_contig\_number\_10352SRS147088\_contig\_number\_304
species
13

1
species

SRS1041138\_contig\_number\_12932
28119
